# Clinicopathological and Prognostic Significance of CBX3 Expression in Human Cancer: a Systematic Review and Meta-analysis

**DOI:** 10.1155/2020/2412741

**Published:** 2020-11-12

**Authors:** Hexin Lin, Xin Zhao, Lu Xia, Jiabian Lian, Jun You

**Affiliations:** ^1^Department of Gastrointestinal Surgery, The First Affiliated Hospital of Xiamen University, Xiamen, China; ^2^Department of Colorectal Surgery, The First Affiliated Hospital of Fujian Medical University, Fuzhou, China; ^3^Laboratory of Cancer Center, The First Affiliated Hospital of Xiamen University, Xiamen, China; ^4^Department of Medical Oncology, The First Affiliated Hospital of Xiamen University, Xiamen, China; ^5^School of Clinical Medicine, Fujian Medical University, Fuzhou, China; ^6^Department of laboratory medicine, Xiamen Key Laboratory of Genetic Testing, The First Affiliated Hospital of Xiamen University, Xiamen, China

## Abstract

**Background:**

Chromebox protein homolog 3 (CBX3) as a member of the heterochromatin-associated protein 1 (HP1) family has been reported to be overexpressed in human cancer tissues. Numerous studies have shown the relationship between the CBX3 expression and clinicopathological factor or prognosis in malignant tumors, but their results are inconsistent. To address these results, a meta-analysis was described to investigate the prognostic value and clinicopathological significance of CBX3 expression in human malignant neoplasms.

**Methods:**

PubMed, Web of Science, Embase, and Chinese National Knowledge Infrastructure (CNKI) were used to search eligible literatures, including publications prior to September 2019. The role of CBX3 in cancer prognosis and clinicopathological characteristics was assessed by pooled hazard ratios (HRs) and odds ratios (ORs) with 95% confidence intervals (CIs).

**Results:**

Eleven studies with 1682 cancer patients were enrolled in this meta-analysis. This analysis demonstrated that the patients' increased CBX3 expression was significantly associated with poor overall survival (OS) (univariate analysis: HR = 1.81, 95% CI 1.46-2.25; multivariate analysis: HR = 1.95, 95% CI 1.63-2.34). Subgroups analysis by tumor type also indicated that high expression of CBX3 was correlated with poor OS in tongue squamous cell carcinoma (HR = 3.31, 95% CI 2.03-5.39), lung cancer (HR = 1.66, 95% CI 1.21-2.29), genitourinary cancer (HR = 2.03, 95% CI 1.15-3.58), and digestive cancer (HR = 1.48, 95% CI 1.23-1.79). For clinicopathological features, high expression of CBX3 was associated with lymph node metastasis (OR = 2.96, 95% CI 1.42-6.20) and lager tumor size (OR = 1.60, 95% CI 1.12-2.28).

**Conclusion:**

The results of this meta-analysis indicated that CBX3 expression may be a novel biomarker for predicting patient prognosis and clinicopathological parameters in multiple human cancer.

## 1. Introduction

According to the GLOBOCAN in 2018, there are 18.1 million new cancer cases and 9.6 million cancer deaths worldwide each year [1]. Cancer has turned out to be one of the leading causes of human death. Although the current treatment of malignant tumors has made considerable progress, the treatment methods for advanced cancer patients are still limited and inoperable. Therefore, finding prognostic-related biomarkers not only provides an effective predictor of the cancer patient's prognosis but can be a potential therapeutic target after further exploration of the mechanism.

Chromebox protein homolog 3 (CBX3) is a member of the heterochromatin protein 1 family, which is involved in several cellular functions, including transcriptional regulation [2], cell differentiation [3], DNA repair [4, 5], and telomere function [6]. Previous studies have reported that CBX3 is upregulated in a variety of cancer tissues, covering colorectal cancer, breast cancer, hepatocellular carcinoma, and lung cancer. Furthermore, high CBX3 expression level has been found to be associated with worse prognosis and adverse clinicopathological factors. However, due to the small sample size, discrete outcomes have prevented consensus on the role of CBX3. Thus, we carried out the first systematic review and meta-analysis to evaluate the prognostic value of CBX3 and to investigate whether CBX3 could be a predictive marker for prognosis and clinicopathological parameters.

## 2. Methods

### 2.1. Literature Search Strategy

PubMed, Web of Science, Embase, and Chinese National Knowledge Infrastructure(CNKI) were used to search included literatures. The search items used were as follows: “CBX3 or HP1*γ* or chromobox 3 or chromobox protein homolog 3 or heterochromatin protein 1 gamma or HP1 gamma” and “cancer or tumor or carcinoma or neoplasm” and “survival or outcome or prognosis.” The reference list in an identified study was also screened manually to acquire other eligible articles. The extracted study was published before September 2019.

### 2.2. Selection Criteria

The inclusion criteria were listed as follows: (a) the expression level of CBX3 was measured by immunohistochemistry (IHC) in primary cancer tissues; (b) literatures which contained information of the CBX3 expression with overall survival (OS) of cancer patients or clinicopathological features such as tumor size, differentiation, lymph node metastasis, and distant metastasis; and (c) papers with sufficient data provided to assess odds ratios (ORs), hazard ratios (HRs), and 95% confidence intervals (CIs). The exclusion criteria included (a) papers without adequate relevant data to estimate HRs or ORs and (b) review articles, letters, case reports, or expert consensus.

### 2.3. Data Extraction and Quality Assessment

Two independent authors scanned all candidate manuscripts based on the inclusion and exclusion criteria. The information and data from each eligible study were extracted by these authors, including the first author's name, year of publication, country, cancer type, sample size, gender, detection measures, analysis type, cutoff value for CBX3 high expression, HR, and OR with 95% CIs. Each included article was scored by the Newcastle-Ottawa scale (NOS) to assess the quality [7]. A study with a NOS score ≥ 6 was considered methodologically sound and included in the final analysis. Any disagreement between these two authors was resolved by obtaining a consensus with third authors.

### 2.4. Statistical Analysis

Data were analyzed using the RevMan 5.3 software and STATA15.1. When the HR values were not directly reported, we obtained additional data from the original authors. When the request was not answered, the HR values were extracted from Kaplan–Meier curves by the Engauge Digitizer 4.1 software. Heterogeneity was calculated by the chi-squared test and *I*-squared statistics. If *I*^2^ ≥ 50% and *P* ≤ 0.10 both establish, meta-analysis used a random-effects model; otherwise, a fixed-effects model was selected. In addition, subgroup analysis and sensitivity analysis were used to minimize the influence of heterogeneity. Publication bias was estimated qualitatively using Begg's and Egger's tests with funnel plots. If Begg's and Egger's results indicated that the publication bias exists, the trim and fill method was used to examine the sensitivity of the result [8]. A difference was considered statistically significant if two-sided *P* < 0.05.

### 2.5. Review Registration

This review's protocol was registered in PROSPERO (CRD42020150946).

## 3. Results

### 3.1. Study Characteristics

**Figure 1:**
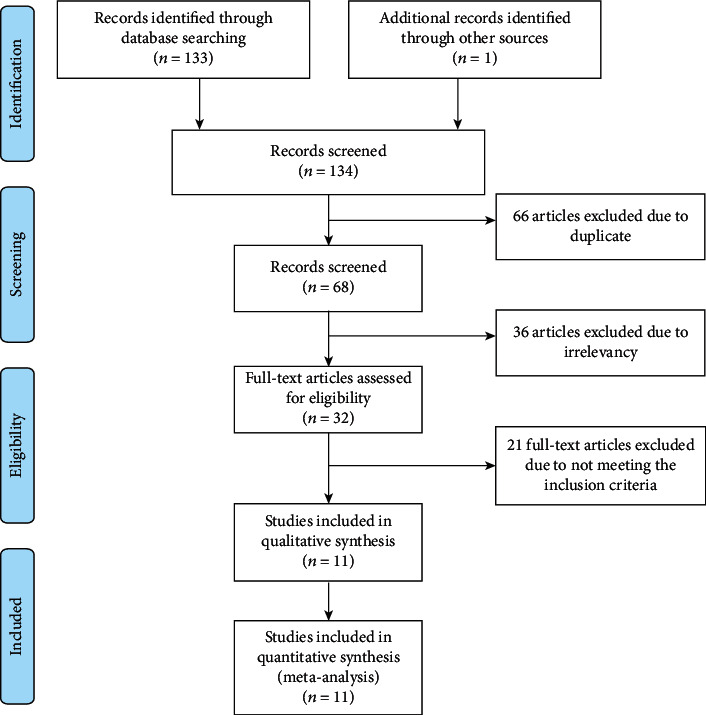
Flowchart of literature retrieval and study selection.

This meta-analysis included 11 eligible articles with a total of 1682 cancer patients. The literature inclusion flow chart is illustrated in Figure 1. The main characteristics of the included literatures were exhibited in Table 1 and Supplementary Table 1. All patients were divided into two cohorts by the level of CBX3 expression and IHC was used for detection. Eight different types of cancer were involved in this meta-analysis, including tongue squamous cell carcinoma (TSCC) [9, 10], colorectal cancer (CRC) [11, 12], non-small-cell lung cancer (NSCLC) [13, 14], renal cancer (RC) [15], prostate cancer (PCa) [16], bladder urothelial carcinoma (BLCA) [17], hepatocellular carcinoma (HCC) [18], and cervical cancer (CESC) [19].

### 3.2. Association between CBX3 Expression and OS

**Figure 2:**
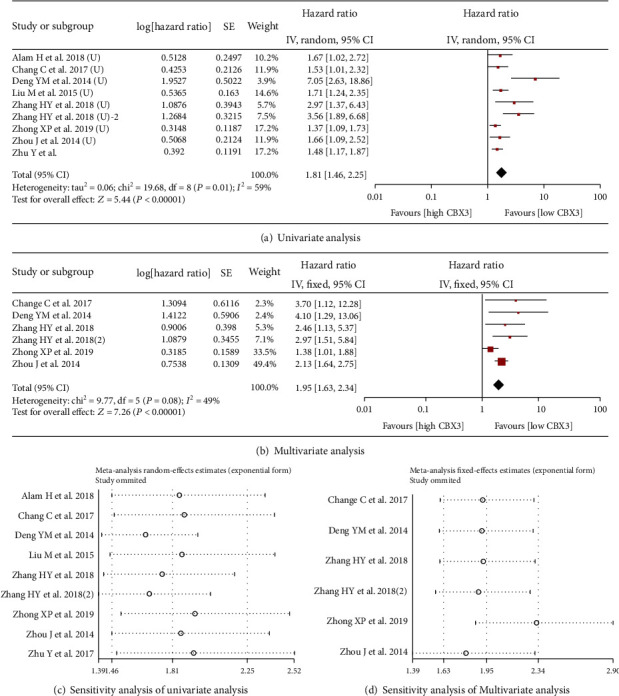
Forest plots for the association between CBX3 expression and OS with (a) univariate analysis and (b) multivariate analysis in cancer patients. (c), (d) Sensitivity analysis of univariate analysis and multivariate analysis of OS, respectively. Abbreviations: SE: standard error; CI: confidence interval; IV: inverse variance; OS: overall survival.

**Figure 3:**
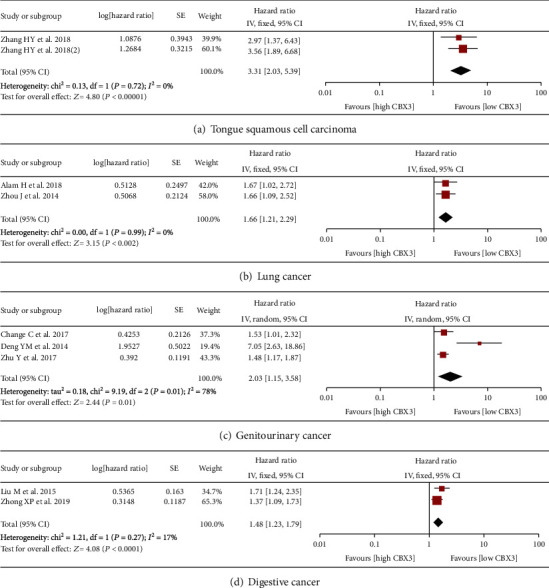
Forest plots for subgroup analysis of OS (univariate analysis) by CBX3 expression in various cancer types: (a) tongue squamous cell carcinoma, (b) lung cancer, (c) genitourinary cancer, (d) digestive cancer. Abbreviations: SE: standard error; CI: confidence interval; IV: inverse variance; OS: overall survival.

There were 9 studies and 6 studies that reported OS data with univariate analysis and multivariate analysis, respectively. As shown in Figure 2(a), high expression of CBX3 in univariate analysis correlated with shorter overall survival times in patients with malignant tumors (HR = 1.81, 95% CI 1.46-2.25, *P* < 0.00001) and had the same result in multivariate analysis (HR = 1.95, 95% CI 1.63-2.34, *P* < 0.00001) (Figure 2(b)). The result of univariate analysis displayed significant heterogeneity (*I*^2^ = 59%, *P* = 0.01), and further subgroup analysis was performed according to cancer species to explore the source of heterogeneity. Stratified analysis showed that high expression of CBX3 was significantly correlated with poor prognosis of tongue squamous cell carcinoma (HR = 3.31, 95% CI 2.03-5.39, *P* < 0.00001), lung cancer (HR = 1.66, 95% CI 1.21-2.29, *P* = 0.002), genitourinary tumors (HR = 2.03, 95% CI 1.15-3.58, *P* = 0.01), and digestive cancer (HR = 1.48, 95% CI 1.23-1.79, *P* < 0.0001) (Figure 3). In addition, sensitivity analysis showed that the meta-analyses of OS were stable (Figure 2(c) and 2(d)).

### 3.3. Association between CBX3 Expression and Clinicopathological Features

**Figure 4:**
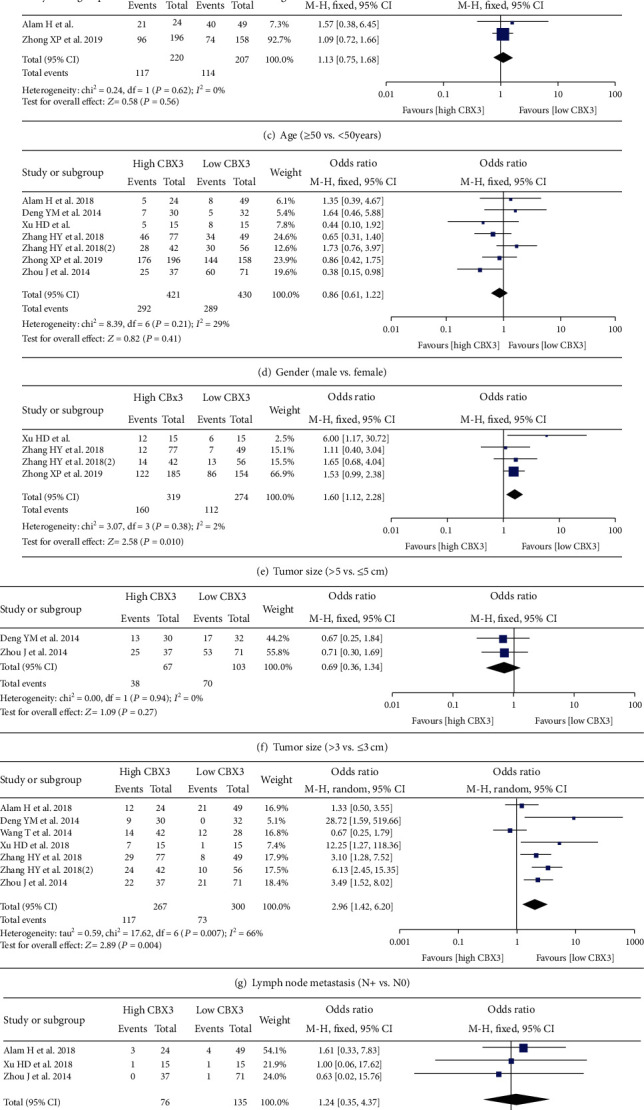
Forest plots for the association between CBX3 expression and in cancer patients: (a) age (≥65 vs. <65 years); (b) age (≥60 vs. <60 years); (c) age (≥50 vs. <50years); (d) gender (male vs. female); (e) tumor size (>5 vs. ≤5 cm); (f) tumor size (>3 vs. ≤3 cm); (g) lymph node metastasis (N+ vs. N0); (h) distant metastasis (M+ vs. M0); (i) degree of differentiation (well+moderate vs. poor). Abbreviations: SE: standard error; CI: confidence interval; IV: inverse variance; OR: odds ratio.

There were seven studies with 851 patients that reported clinicopathological data grouped by CBX3 expression level. The results revealed that a high expression level of CBX3 was apparently related to lymph node metastasis (N+ vs. N0, OR = 2.96, 95% CI 1.42-6.20, *P* = 0.004); further sensitivity analysis showed that this result was reliable (Supplementary Figure 1). In contrast to the low CBX3 expression group, the tumor size was significantly larger in the high CBX3 expression group (>5 vs. ≤5 cm, OR = 1.60, 95% CI 1.12-2.28, *P* = 0.01). The relevant results showed that the CBX3 expression level was not significantly associated with age (≥65 vs. <65 years, OR = 1.51, 95% CI 0.84-2.69, *P* = 0.17; ≥60 vs. <60 years, OR = 0.65, 95% CI 0.37-1.14, *P* = 0.13; ≥50 vs. <50 years, OR = 1.13, 95% CI 0.75-1.68, *P* = 0.56), gender (male vs. female, OR = 0.97, 95% CI 0.89-1.05, *P* = 0.41), tumor size (>3 vs. ≤3 cm, OR = 0.69, 95% CI 0.36-1.34, *P* = 0.27), distant metastasis (M+ vs. M0, OR = 1.24, 95% CI 0.35-4.37, *P* = 0.74), and differentiation (well+moderate vs. poor, OR = 0.97, 95% CI 0.64-1.49, *P* = 0.9) (Table 2 and Figure 4).

### 3.4. Publication Bias

**Figure 5:**
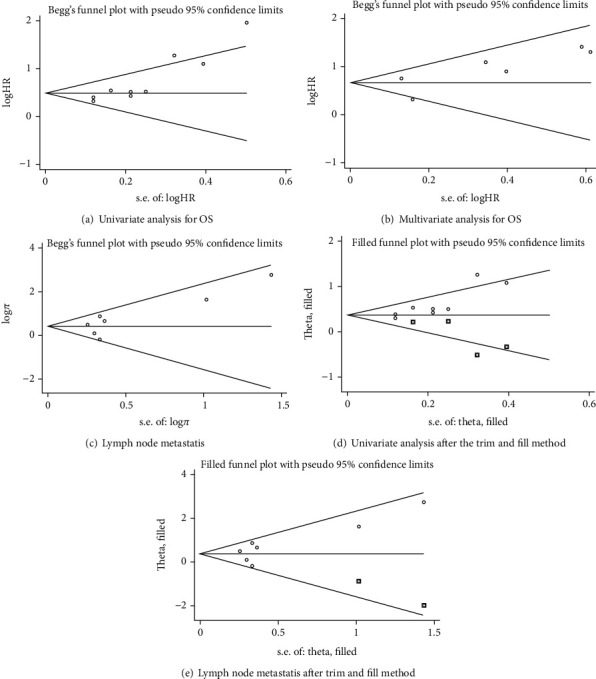
(a), (b), (c) Begg's funnel plot estimation of the publication bias for OS and lymph node metastasis. (d), (e) Begg's funnel plot of univariate analysis and lymph node metastasis for OS after trim and fill. Abbreviations: OS: overall survival.

This meta-analysis adopted Begg's test and Egger's test to evaluate publication bias. There was no significant publication bias in the multivariate analysis of the relationship between the CBX3 expression and OS (Begg's test *P* = 0.452, Egger's test *P* = 0.173, Figure 5(b)), while there was a significant publication bias in the univariate analysis (Begg's test *P* = 0.009, Egger's test *P* = 0.001, Figure 5(a)). The published bias graph after the trim and fill method was symmetric, and the meta-analysis results did not change (HR = 1.47, 95% CI 1.18-1.84, *P* = 0.001, Figure 5(c)), indicating that the results were stable and credible. In addition, we found high heterogeneity in the meta-analysis of lymph node metastasis, but Begg's and Egger's tests showed no significant publication bias (Begg's test *P* = 0.230, Egger's test *P* = 0.149) (Figure 5(d), 5(e)).

## 4. Discussion

With the increase in the incidence of cancers, humans have never stopped exploring effective treatments and prognostic biomarkers of malignant tumors. In recent years, many studies have described that CBX3 is upregulated in various malignant tumors and is closely related to the prognosis of cancer patients. However, whether CBX3 is suitable as a clinicopathological marker or prognostic marker remains questionable. This meta-analysis was designed to explore the relationship between CBX3 expression and clinical data in patients with malignant tumors.

The clinical data of this meta-analysis were collected from 11 studies of 1682 patients with malignant tumors. Our results indicated that increased CBX3 expression in malignancies is significantly associated with poor survival. The results were consistent with those of multivariate analysis and univariate analysis. The TCGA database also showed that the mRNA level of CBX3 was significantly correlated with the overall survival time of patients with pancreatic cancer [20], hepatocellular carcinoma [21], prostate cancer [16], and glioma [22, 23]. CBX3 has been reported to promote the proliferation, invasion, and migration of tumor cells [16, 18, 20, 22, 24]. In our analysis of clinicopathological data, the high expression of CBX3 was indeed associated with larger tumor size and lymph node metastasis in cancer patients. Previous studies have reported that CBX3 plays a certain role in cell differentiation, and the downregulation of CBX3 can promote cell differentiation [3]. However, some studies hold the opposite view [25]. The results of this meta-analysis manifested that CBX3 had no significant effect on the tumor cell differentiation.

In terms of the cell cycle, CBX3 has been proved to promote G1/S cell cycle transition in tongue squamous cell carcinoma and colon cancer and has been shown to arrest the cell cycle in the G2/M phase in malignant gliomas and pancreatic cancers [9, 10, 20, 22, 26]. Ma et al. [27] presented that CBX3 knockdown in osteosarcoma promotes apoptosis and arrests the cell cycle in G0 and G1 phases. In terms of the regulation of gene expression by microRNA, mir-30a, mir-30b, and mir-320a exert anticancer effects by inhibiting CBX3 expression in colorectal cancer and esophageal squamous cell carcinoma [11, 28, 29]. Chang et al. [16] proved that HP1*γ*/miR-451a/c-Myc regulatory circuitry exists in PCa cells and plays a vital role in PCa progression. In terms of tumor metabolism, CBX3 has been verified to be involved in the anaerobic glycolysis of colorectal cancer cells [30]; Chen et al. [31] showed that CBX3 can promote the proliferation of pancreatic cancer by inhibiting the negative regulator of aerobic glycolysis FBP1. Sun et al. [32] reported that the downregulated CBX3 expression can enhance the tumor-killing ability of CD8+T cells. In patients with nonsmall cell lung cancer, CBX3 expression has a significant correlation with EGFR mutations [33]. And in non-small-cell lung cancer tumor-initiating cells, CBX3 and H3K9me3 are significantly increased and inhibited DNA damage related to antineoplastic therapy efficacy [34]. It should be noted that CBX3 not only plays a crucial role in the development and progression of malignant tumors but also becomes a reliable prognostic indicator and potential target therapeutic site for cancer patients.

However, there were some deficiencies and limitations in this meta-analysis. First, some included articles provided incomplete survival data and can only be extracted from the Kaplan-Meier survival curve. Second, there were differences in the cutoff values for evaluating the high expression of CBX3 in the article included in this meta-analysis. Thirdly, the heterogeneity existed in the meta-analysis of OS for univariate analysis and lymph node metastasis. Considering the possibility of being affected by different cancer types, a subgroup analysis and random effects model were performed to deal with heterogeneity. Finally, there was a publication bias in this meta-analysis, because the articles with negative results are less likely to be published. The trim and fill method was used to verify that the publication bias did not affect the results.

In conclusion, existing studies have demonstrated that CBX3 is highly expressed in a variety of cancers and predicts a poor prognosis for malignancy. After more in-depth mechanism researches, CBX3 is expected to be an effective prognostic biomarker and therapeutic target for cancer patients.

## Figures and Tables

**Table 1:** Characteristics of included studies in the meta-analysis.

Study	Year	Country	Cancer type	Case no.	Male/female	Detection method	Cutoff value	Increased CBX3 (%)	HR for OS (U)	HR for OS (M)	NOS. (scores)
Zhong et al.	2019	China	HCC	354	34/320	IHC	Youden's index	196 (55.4%)	1.37^∗^	1.38	9
Zhang et al.	2018	China	TSCC	126	81/45	IHC	Score > 4	77 (61.1%)	2.97	2.46	9
Zhang et al.	2018	China	TSCC	98	58/40	IHC	Score > 6	42 (42.9%)	3.56	2.97	8
Xu et al.	2018	China	CRC	30	17/13	IHC	Score > 6	15 (50.0%)	NA	NA	9
Alam et al.	2018	America	LUAD	73	13/60	IHC	Score > 4	24 (32.8%)	1.67^∗^	NA	8
Chang et al.	2017	China	PCa	62	62/0	IHC	Mean	34 (54.8%)	1.53	3.7	7
Zhu et al.	2017	China	RCC	521	NA	IHC	NA	259 (49.7)	1.48	NA	6
Liu et al.	2015	China	CRC	178	104/74	IHC	Score > 8.94	103 (61.2%)	1.71^∗^	NA	9
Deng et al.	2014	China	BLCA	62	12/50	IHC	Score > 5	30 (48.4%)	7.05	4.1	9
Zhou et al.	2014	China	NSCLC	108	85/23	IHC	Staining > 40%	30 (27.8%)	1.66	2.13	9
Wang et al.	2014	China	CESC	70	0/70	IHC	Staining > 10%	42 (60.0%)	NA	NA	7

Abbreviations: IHC: immunohistochemistry; OS: overall survival; NOS: Newcastle-Ottawa Scale; U: univariate analysis; M: multivariate analysis; NA: not available; HCC: hepatocellular carcinoma; TSCC: tongue squamous cell carcinoma; CRC: colorectal cancer; LUAD: lung adenocarcinoma; PCa: prostate cancer; RCC: renal carcinoma; BLCA: bladder urothelial carcinoma; NSCLC: non-small-cell lung cancer; CESC: cervical cancer. ^∗^The HR values were extracted by the Engauge Digitizer 4.1 Software.

**Table 2:** OR for the relationship between positive CBX3 expression and clinicopathological features.

Categories	Studies no.	Case no.	Pooled OR (95% CI)	Model	Heterogeneity	
					*I* ^2^	*P* value
Age (≥65 vs. <65years)	3	200	1.51 (0.84, 2.69)	Fixed	0%	0.81
Age (≥60 vs. <60years)	2	224	0.65 (0.37, 1.14)	Fixed	0%	0.76
Age (≥50 vs. <50years)	2	427	1.13 (0.75, 1.68)	Fixed	0%	0.62
Gender (male vs. female)	7	851	0.86 (0.61, 1.22)	Fixed	29%	0.21
Tumor size (>5 vs. ≤5 cm)	4	593	1.60 (1.12, 2.28)	Fixed	2%	0.38
Tumor size (>3 vs. ≤3 cm)	2	170	0.69 (0.36, 1.34)	Fixed	0%	0.94
Lymph node metastasis (N+ vs. N0)	7	567	2.96 (1.42, 6.20)	Random	66%	0.007
Distant metastasis (M+ vs. M0)	3	211	1.24 (0.35, 4.37)	Fixed	0%	0.86
Degree of differentiation (well+moderate vs. poor)	5	645	0.97 (0.64, 1.49)	Fixed	0%	0.76

Abbreviations: OR: odds ratio.

## Supplementary Materials

Supplementary Materials Supplementary Figure 1. Sensitivity analysis of lymph node metastasis. Supplementary Table 1. Characteristics of the clinicopathological features. HCC: hepatocellular carcinoma, TSCC: tongue squamous cell carcinoma, CRC: colorectal cancer, LUAD: lung adenocarcinoma, PCa: prostate cancer, RCC: renal carcinoma, BLCA: bladder urothelial carcinoma, NSCLC: non-small cell lung cancer, CESC: cervical cancer. NA: not available, # There are missing cases here. ∗ The values were extracted by Engauge Digitizer 4.1
