# Supplementary material for: Clinicopathological and Prognostic Significance of CBX3 Expression in Human Cancer: a Systematic Review and Meta-analysis
Source: Dis Markers. 2020 Nov 12;2020:2412741. doi: 10.1155/2020/2412741 (PMC7676940; doi:10.1155/2020/2412741)
Supplement: Supplementary Materials — Supplementary Figure 1. Sensitivity analysis of lymph node metastasis. Supplementary Table 1. Characteristics of the clinicopathological features. HCC: hepatocellular carcinoma, TSCC: tongue squamous cell carcinoma, CRC: colorectal cancer, LUAD: lung adenocarcinoma, PCa: prostate cancer, RCC: renal carcinoma, BLCA: bladder urothelial carcinoma, NSCLC: non-small cell lung cancer, CESC: cervical cancer. NA: not available, # There are missing cases here. ∗ The values were extracted by Engauge Digitizer 4.1 [file 2412741.f1.zip › Supplementary Table 1.docx]

**Supplementary Table 1.** Characteristics of the clinicopathological features.

HCC: hepatocellular carcinoma, TSCC: tongue squamous cell carcinoma, CRC: colorectal cancer, LUAD: lung adenocarcinoma, PCa: prostate cancer, RCC: renal carcinoma, BLCA: bladder urothelial carcinoma, NSCLC: non-small cell lung cancer, CESC: cervical cancer. NA: not available, # There are missing cases here. * The values were extracted by Engauge Digitizer 4.1 Software.

| Study | Cancer Type | Surgery | Metastasis | T stage (I+II/III+IV) | N stage (N0/N+) | Differentiation  (H/L) | TNM stage (I+II/III+IV) | Follow up time |
| --- | --- | --- | --- | --- | --- | --- | --- | --- |
|  |  |  |  |  |  |  |  |  |
| Zhong XP et al. | HCC | Yes | Yes | 114/224(I/II-IV) | NA | 36/294^#^ | 253/87 | 2-155(63.5) |
| Zhang HY et al. | TSCC | Yes | Yes | 107/19 | 89/37 | 99/27 | 80/46 | 6.5-97.8(67.6) |
| Zhang HY et al. | TSCC | Yes | Yes | 71/27 | 63/34 | 80/18 | 48/50 | 5-98 |
| Xu HD et al. | CRC | Yes | Yes | 5/25 | 22/8 | 5/25 | 20/10 | NA |
| Alam H et al. | LUAD | Yes | Yes | 66/7 | 40/33 | NA | 49/24 | 3-153 |
| Chang C et al. | PCa | Yes | Yes | 45/16 | 55/6^#^ | 22/28(Gleason Scor 2-7/8-10) | NA | 3.3-107.9* |
| Zhu Y et al. | RCC | Yes | No | NA | NA | NA | NA | 3-125* |
| Liu M et al. | CRC | Yes | NA | NA | NA | 140/38 | NA | 1.5-96* |
| Deng YM et al. | BLCA | Yes | NA | 33/29(NMI/MI) | 53/9 | 21/41 | NA | 1.5-108.3*(60.3) |
| Zhou J et al. | NSCLC | Yes | Yes | NA | 65/43 | NA | 91/17 | 3-83* |
| Wang T et al. | CESC | Yes | No | NA | 44/26 | NA | 25/45(IA-IIA/IIB-IIIB) | NA |
