# Supplementary figures and images for: Clinicopathological and Prognostic Significance of CBX3 Expression in Human Cancer: a Systematic Review and Meta-analysis
Source: Dis Markers. 2020 Nov 12;2020:2412741. doi: 10.1155/2020/2412741 (PMC7676940; doi:10.1155/2020/2412741)

**Supplementary Figure 1. Sensitivity analysis of lymph node metastasis.**

**
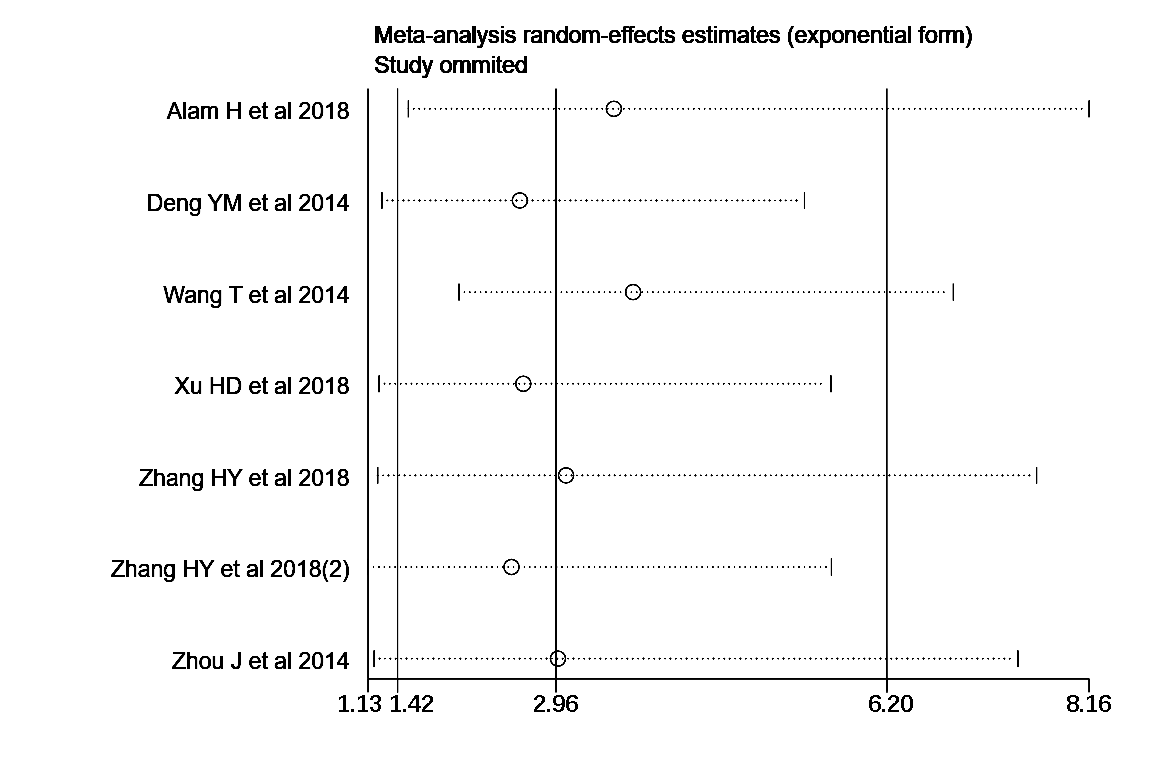
**

Supplement: Supplementary Materials — Supplementary Figure 1. Sensitivity analysis of lymph node metastasis. Supplementary Table 1. Characteristics of the clinicopathological features. HCC: hepatocellular carcinoma, TSCC: tongue squamous cell carcinoma, CRC: colorectal cancer, LUAD: lung adenocarcinoma, PCa: prostate cancer, RCC: renal carcinoma, BLCA: bladder urothelial carcinoma, NSCLC: non-small cell lung cancer, CESC: cervical cancer. NA: not available, # There are missing cases here. ∗ The values were extracted by Engauge Digitizer 4.1 [file 2412741.f1.zip › Supplementary Figure1.docx]
